# Modelling Lowe syndrome and Dent-2 disease using zebrafish

**DOI:** 10.3389/fcell.2025.1637005

**Published:** 2025-07-24

**Authors:** Martin Lowe

**Affiliations:** Faculty of Biology, Medicine and Health, University of Manchester, Manchester, United Kingdom

**Keywords:** zebrafish, Lowe syndrome, Dent-2 disease, neurological, renal, endocytosis, ciliogenesesis

## Abstract

Lowe syndrome and Dent-2 disease are caused by mutations in the gene encoding OCRL, an inositol 5-phosphatase. The phenotype manifests in the eyes, brain and kidney, with the extra-renal features milder in the case of Dent-2 disease. Zebrafish has been used to study OCRL function *in vivo* and to successfully model these two rare genetic conditions. OCRL-deficient zebrafish have neurodevelopmental defects, which may lie downstream of disrupted endosomal trafficking or primary cilia function. OCRL-deficient zebrafish also have a renal tubular phenotype, with defective endocytosis, abnormal lysosomal function, and shortening of the renal tubule. These defects can account for the low molecular weight proteinuria seen in Lowe syndrome and Dent-2 disease and may explain the other renal features seen in both conditions. Chemical and genetic rescue experiments indicate that zebrafish can be used to test potential therapeutic approaches for Lowe syndrome and Dent-2 disease, raising the possibility of a phenotypic screen for these conditions in zebrafish. Alongside other models, zebrafish has proven its worth in studying Lowe syndrome and Dent-2 disease and should continue to serve as a valuable model going forwards.

## Introduction

Lowe syndrome is a rare X-linked disorder (incidence is estimated at approximately 1:500000–1:1000000) that manifests primarily in the eyes, brain and kidneys, with common clinical features of congenital cataracts, glaucoma, hypotonia, seizures, intellectual disability, delayed development and proximal renal tubulopathy (Lewis et al., 1993; Nussbaum and Suchy, 2019). The renal phenotype is progressive and can lead to loss of glomerular filtration and ultimately renal failure. Lowe syndrome was discovered in the 1950’s (Lowe et al., 1952), but it was not until the early 1990’s that the causative gene was identified as OCRL (Attree et al., 1992), which encodes an inositol 5-phosphatase that has been extensively studied since, and which I describe further below. More recently, mutations in OCRL were also shown to cause Dent-2 disease (Hoopes et al., 2005), which shares the same renal pathology as Lowe syndrome but in which the extra-renal symptoms are either absent or milder than those seen in Lowe syndrome (Bokenkamp et al., 2009). Dent-2 disease is so named to distinguish it from the originally identified Dent disease, a renal disorder caused by mutations in the endosomal chloride channel Clc-5 (Devuyst and Thakker, 2010). The mechanism by which mutations in the same gene, OCRL, give rise to different patient phenotypes remains to be determined. Dent-2 mutations tend to cluster in the first seven exons of OCRL (Shrimpton et al., 2009; Hichri et al., 2011; Gianesello et al., 2021), and it has been proposed that alternative splicing of OCRL, which can give rise to a shorter form starting at exon 8, may explain how different phenotypes can arise in patients (Shrimpton et al., 2009; Sakakibara et al., 2022). However, genetic background is also a likely factor considering that in some cases the same mutation in OCRL can result in either Dent-2 disease or Lowe syndrome, and Lowe syndrome of varying severity can also result from the same mutation in OCRL (Hichri et al., 2011; Montjean et al., 2015; Gianesello et al., 2021). There is no cure for Lowe syndrome or Dent-2 disease, with current interventions aimed at managing the symptoms of each condition. For example, congenital cataracts in Lowe syndrome can be removed by surgery, and supplementation can help manage the consequences of renal dysfunction in Lowe syndrome and Dent-2 disease patients (Lewis et al., 1993; Nussbaum and Suchy, 2019). Over recent years our understanding of OCRL cellular function has improved markedly, and animal models have been developed to investigate the mechanisms underlying Lowe syndrome and Dent-2 disease (Loovers et al., 2007; Bothwell et al., 2011; Ramirez et al., 2012; Cheng et al., 2015; Del Signore et al., 2017). In this review I will discuss the use of zebrafish as an animal model to better understand the roles of OCRL *in vivo* and the pathology of Lowe syndrome and Dent-2 disease. I also discuss how zebrafish may be used to develop new therapeutic strategies for these conditions.

## OCRL structure and function

OCRL is well studied, and its structure and cellular functions have been reviewed extensively elsewhere (Mehta et al., 2014; De Matteis et al., 2017; de Sa et al., 2025). Hence, I will only briefly describe them here. OCRL is comprised of several conserved domains, namely, an N-terminal pleckstrin homology (PH), central 5-phosphatase, and C-terminal ASH and RhoGAP-like domains. The central 5-phosphatase domain preferentially hydrolyses PI(4,5)P_2_ to PI4P, which can regulate PI(4,5)P_2_-dependent cellular processes. OCRL interacts with various binding partners including components of the clathrin vesicle trafficking machinery (clathrin heavy chain, clathrin adaptor AP2), several Rab GTPases, the endosomal adaptor proteins IPIP27A and B (also called Ses1/2, FAM109A/B, PHETA1/2) and APPL1, and the Rho GTPases Cdc42 and Rac1. These interactions help target OCRL to specific subcellular locations where it can regulate different cellular processes. Functional studies in cells have implicated OCRL in phagocytosis and macropinocytosis, clathrin-dependent endocytosis and endosomal trafficking, ciliogenesis, cytokinesis, cell adhesion and migration and lipid homeostasis. Its role in these processes has been covered elsewhere (Mehta et al., 2014; De Matteis et al., 2017; de Sa et al., 2025). Importantly, in vertebrates OCRL has a paralogue called INPP5B with which it shares significant sequence identify, the same domain organisation and many common binding partners (de Sa et al., 2025). INPP5B is less well studied than OCRL but likely shares several of the functional roles of OCRL. This aspect has been covered in greater detail elsewhere (de Sa et al., 2025).

## Zebrafish as a disease model

Zebrafish (*Danio rerio*) is a small freshwater teleost native to the southern Himalayas. Since the 1980’s zebrafish has emerged as an excellent vertebrate model to study development and disease. Zebrafish shares a high degree of genetic similarity to humans with approximately 70% of all human genes having at least one orthologue in zebrafish (Howe et al., 2013). Most developmental processes and organ systems are also well conserved between zebrafish and human (Kimmel, 1989). Zebrafish have high fecundity, producing several hundred embryos per mating, the embryos develop externally, and development is rapid (organogenesis is complete within 24 h), making it an excellent model for the study of developmental processes (Kimmel, 1989). Zebrafish embryos are transparent, which makes them suitable for imaging by light microscopy (Antinucci and Hindges, 2016). Zebrafish are also amenable to a variety of genetic approaches to generate transgenic reporter lines to study protein localisation and developmental or physiological processes of interest, or to introduce mutations that disrupt gene function. These include ENU-based mutagenesis, Tol2-mediated transgenesis, and as more commonly used these days CRISPR-Cas9-based gene editing (Grunwald and Streisinger, 1992; Kwan et al., 2007; Varshney et al., 2015b). The latter can be used in a transient manner to generate *de novo* knockout animals in the F0 generation (crispants), or to make stable genetic knock-out lines (Hwang et al., 2013; Wu et al., 2018; Kroll et al., 2021). CRISPR technology can also be used in zebrafish to knockin patient mutations, achieved using homology-directed repair or base editing for the precise introduction of point mutations into genes of interest (Liu et al., 2025). This genetic tractability, combined with the other advantages outlined above, makes zebrafish suitable for modelling a wide variety of genetic conditions (Lieschke and Currie, 2007; Choi et al., 2021). Moreover, because of the large numbers of offspring produced by zebrafish, and their suitability for imaging, zebrafish offers the opportunity for phenotypic screening. This can be used in the context of drug screens to identify compounds that rescue a morphological or functional phenotype (MacRae and Peterson, 2015; Patton et al., 2021), providing it can be scored easily, or the possibility of doing genetic screens to identify new players in processes of interest (Driever et al., 1996; Haffter et al., 1996; Varshney et al., 2015a). Finally, from an ethical point of view, it is worth considering that zebrafish offer advantages in terms of the 3Rs, for example, allowing the replacement of mammalian species, and the reduction in animal use overall; within the EU and UK zebrafish are not considered protected animals until 5 days post-fertilisation. At this stage development is complete and most physiological processes are occurring as seen in adulthood. Hence, experiments can be performed at larval stages at which point the larvae are not classed as protected animals, thereby reducing animal usage.

As with any model, there are also some disadvantages of using zebrafish. Although most genes are conserved between humans and fish, not all are, and in some cases their functions may have evolved differently. The teleost lineage underwent a whole genome duplication event around 320 million years ago, meaning that ∼20%–30% of all genes in the current zebrafish genome have duplicates (Howe et al., 2013). This duplication means that loss-of-function mutations in one gene copy may be compensated by its paralogue, complicating interpretation of knockout phenotypes (Tasnim et al., 2024). Differences are also apparent at the anatomical level, meaning that zebrafish is not suited to studying certain organ systems. However, despite these limitations, zebrafish remain an excellent model for studying developmental and disease processes.

## Zebrafish as a model for Lowe syndrome and Dent-2 disease

OCRL is highly conserved between humans and zebrafish, sharing 65% identity at the level of the coding DNA sequence and 60% identity at the protein should be level (Ramirez et al., 2012). The functional domains of OCRL are also well conserved between humans and zebrafish, as are the binding sites for the known OCRL interaction partners (Ramirez et al., 2012). Similarly, the OCRL paralogue INPP5B is also well conserved between human and zebrafish, although the C-terminal CAAX box that is prenylated in humans is not conserved in zebrafish (de Sa et al., 2025). This contrasts with ‘lower’ eukaryotes, where there is only one paralogue, named dOCRL in *Drosophila melanogaster*, OCRL-1 in *Caenorhabditis elegans*, and Dd5P4 in *Dictyostelium discoideum* (de Sa et al., 2025). Each of these paralogues is equally similar to vertebrate OCRL and INPP5B. Importantly, neither OCRL nor INPP5B are duplicated in zebrafish compared to mammals. The interaction partners of OCRL and INPP5B are also well conserved between human and zebrafish, which includes the presence of orthologues of both IPIP27A and IPIP27B, which contrasts to ‘lower’ eukaryotes where, like OCRL and INPP5B, there is only paralogue. OCRL tissue expression in zebrafish is similar to that observed in mammals, with widespread expression across all tissues and relative enrichment in the brain (Ramirez et al., 2012). The tissue-specific splicing of OCRL is also conserved between mammals and zebrafish, with the isoforms termed ‘a’ and ‘b’, generated by differential splicing of exon 18 to include or exclude an eight amino acid segment important for clathrin binding respectively, showing the same distribution across tissues; the longer isoform ‘a’ is the only form in brain, whereas isoform ‘b’ is the most abundant form in most other tissues (Johnson et al., 2003; Choudhury et al., 2009; Ramirez et al., 2012). It was also shown that OCRL is expressed throughout early zebrafish development, consistent with a role during embryogenesis (Ramirez et al., 2012). Together, these observations indicate excellent conservation of OCRL between humans and zebrafish, supporting its use as a model for Lowe syndrome and Dent-2 disease.

### Modelling the disease phenotype

The first zebrafish model to study Lowe syndrome and Dent-2 disease was generated using a genetrap approach, resulting in a zebrafish mutant containing a retroviral insertion close to the OCRL promoter (Ramirez et al., 2012) (Table 1). This insertion results in attenuated expression of OCRL, reducing OCRL protein levels by ∼70%. A commonly used complementary approach has been to knock-down OCRL expression using morpholinos. These anti-sense oligonucleotides attenuate expression either by blocking translation via targeting of the start codon or preventing mRNA splicing by blocking splice acceptor or donor sites. Translation and splice blocking morpholinos have been used in studies to knock-down OCRL expression in zebrafish (Coon et al., 2012; Luo et al., 2012; Ramirez et al., 2012; Rbaibi et al., 2012; Oltrabella et al., 2015) (Table 1). An ENU-generated zebrafish OCRL mutant has also been used in one study to study OCRL function during neural development (Williams et al., 2022) (Table 1). Finally, zebrafish are highly amenable to CRISPR-Cas9-mediated mutagenesis to generate knockout animals, although this method has yet to be employed for OCRL.

**TABLE 1:** Zebrafish OCRL models to study Lowe syndrome and Dent-2 disease.

Physiological process	Phenotype	Model used	References
Neurodevelopment	Periventricular lesions, gliosis, febrile seizures, reduced brain size	OCRL genetrap mutant OCRL morpholino	Ramirez et al. (2012)
Neurodevelopment	Endocytic trafficking defects in neuroepithelium	OCRL mutant (ENU-generated)	Williams et al. (2022)
Renal tubular function	Endocytic uptake defect, lysosomal defects	OCRL genetrap mutant OCRL morpholino	Oltrabella et al. (2015), De Leo et al. (2016)
Renal tubular function	Endocytic uptake defect, ciliogenesis defect	OCRL morpholino	Rbaibi et al. (2012)
Renal tubular function	Shortening of renal tubule	OCRL genetrap mutant	Gliozzi et al. (2020)
Embryonic development	Shortened body axis, small eyes, hypopigmentation, ciliogenesis defect	OCRL morpholino	Coon et al. (2012)
Embryonic development	Hydrocephalus, shortened body axis, small eyes, hypopigmentation, ciliogenesis defect	OCRL morpholino	Luo et al. (2012)

### Neurological features

**FIGURE 1:**
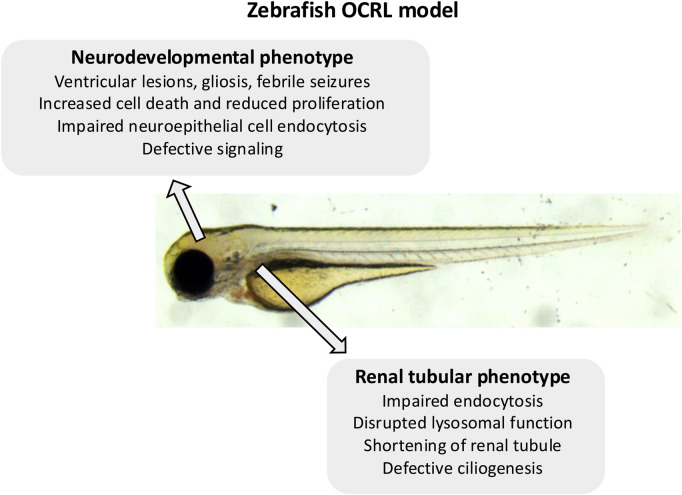
Summary of the zebrafish OCRL model phenotypes. Indicated are the neurological and renal phenotypes observed in the different OCRL-deficient zebrafish models.

Using both the stable OCRL genetrap model and a translation blocking OCRL morpholino, it was shown that attenuation of OCRL expression manifests as several neurological features similar to those seen in Lowe syndrome patients (Ramirez et al., 2012) (Figure 1; Table 1). The OCRL deficient zebrafish have periventricular lesions and gliosis, indicative of neural tissue damage, reduced brain size, and are more prone to febrile seizures. There is also reduced proliferation and increased cell death in the neural tissue. The mechanisms underlying these phenotypes remain to be determined, but rescue experiments indicate that in addition to its 5-phosphatase activity, OCRL binding to clathrin is important for OCRL function during neural development (Ramirez et al., 2012). There is defective Akt signaling upon OCRL deficiency (Ramirez et al., 2012), which based upon other studies, may lie downstream of defective receptor trafficking in the endosomal pathway (Schenck et al., 2008; Fuentealba et al., 2024). A more recent study has shown that OCRL is expressed in the developing zebrafish neuroepithelium where it localises to endosomal compartments (Williams et al., 2022). The neuroepithelial cells represent a progenitor population that are important for neurogenesis (Taverna et al., 2014). They can endocytose various molecules from their apical pole, which includes morphogens such as Sonic Hedgehog (SHH) (Christ et al., 2012; Kur et al., 2014). Deficiency of OCRL, in this case seen in an ENU-generated null mutant, results in altered endosome morphology in zebrafish neuroepithelial cells, which is accompanied by a reduced rate of receptor-mediated endocytosis (Williams et al., 2022) (Figure 1; Table 1). The abundance of the apical multi-ligand receptor LRP2 (also called megalin), which can bind SHH (McCarthy et al., 2002; Christ et al., 2012), is reduced upon loss of OCRL, consistent with defective trafficking in the endosomal system. Defective endosomal trafficking may therefore impact upon neural development at the level of neural progenitor cells, altering their response to different signaling cues including SHH.

Morpholino-induced depletion of OCRL in zebrafish embryos has been shown to disrupt the biogenesis of primary cilia (Coon et al., 2012; Luo et al., 2012; Rbaibi et al., 2012) (Table 1), which play a key role in signaling during neural development (Youn and Han, 2018; Park et al., 2019). Primary cilia act as antennae on neural progenitor cells to mediate responses to different neurodevelopmental cues, including different growth factors and morphogens including SHH, Notch and Wnt (Mill et al., 2023). Hence, defective cilia formation is likely to impact upon neural development in different ways, but differentiation from progenitors is likely to be one of them. This would be consistent with recent studies looking at Lowe patient-derived iPSC cells, where defects in Notch and SHH signaling were correlated with reduced neurogenesis and increased glial cell differentiation (Lo et al., 2024; Sharma et al., 2024). The mechanisms by which loss of OCRL affects ciliogenesis remain to be fully determined, but disruption of trafficking to the cilium is likely involved (Coon et al., 2012). Primary cilia are also important for maintaining healthy eye function, and it has been shown that OCRL-deficient mice are prone to glaucoma through disrupted cilia function (Prosseda et al., 2020). OCRL-deficient zebrafish do not display an overt eye pathology, for example, cataracts, which is a hallmark of Lowe syndrome. However, a more in-depth analysis is required to determine whether other more subtle eye phenotypes are present, including, for example, glaucoma.

### Renal system

**FIGURE 2:**
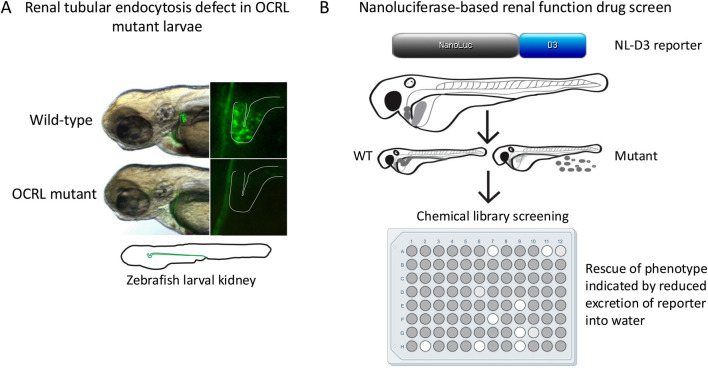
Renal impairment in the OCRL zebrafish model and possible drug screening approach. **(A)** Loss of endocytic tracer uptake in zebrafish OCRL genetrap mutant larvae shown by fluorescence microscopy, indicating defective endocytosis. Note that uptake normally occurs in the proximal region of the larval kidney tubule, as is the case in the mammalian nephron. **(B)** High throughput assay for drug screening in zebrafish larvae. The nanoluciferase (NL)-receptor associated protein D3 region (D3) reporter is expressed in the liver and secreted into the bloodstream, from where it is filtered at the glomerulus and reabsorbed into the proximal tubule by endocytosis. Defective glomerular filtration or renal tubular absorption results in the reporter being excreted into the water, which can be measured by luminescence. The high throughput format allows for chemical screening to identify compounds that reduce urinary excretion of the reporter and rescue the phenotype.

A shared feature of Lowe syndrome and Dent-2 disease is proximal renal tubulopathy, with the hallmark symptom of low molecular weight proteinuria (Bockenhauer et al., 2008; Bokenkamp et al., 2009). It was hypothesised that defective trafficking of LRP2 (and its coreceptor cubilin), which mediates the uptake of low molecular proteins from the renal filtrate into proximal tubule cells (Christensen et al., 2012), may underlie this phenomenon (Norden et al., 2002). The renal system of zebrafish larvae is functionally analogous to that of mammals, but much simpler anatomically with only two nephrons compared to millions in the adult kidney. This conservation of function and simpler morphology makes zebrafish an excellent model to study kidney development and disease (Drummond and Davidson, 2010; Morales and Wingert, 2017; Elmonem et al., 2018). It is possible to assess renal tubular endocytosis in zebrafish larvae, which can be visualized by injecting different fluorescent endocytic tracers and ligands into the bloodstream and visualizing their accumulation within the renal tubules (Drummond et al., 1998; Anzenberger et al., 2006). Using the OCRL genetrap mutant and an OCRL morpholino, it was shown that OCRL-deficiency results in an endocytic defect in the proximal tubule, which is accompanied by altered endosome abundance and reduced levels of LRP2 protein (Oltrabella et al., 2015) (Figures 1, 2A; Table 1). Rescue experiments showed that both OCRL 5-phosphatase activity and its engagement with endocytic machinery is important for its function in the pathway. This is further supported by a follow-up study in which knockout of the OCRL interaction partner IPIP27A, which functions as an endosomal adaptor protein, was shown to phenocopy loss OCRL at the level of endocytic trafficking in the renal tubule (Oltrabella et al., 2021). Rescue experiments also showed that reducing the excess PI(4,5)P_2_ seen in OCRL-deficient zebrafish larvae by knocking down PIP5-kinase could restore renal tubular endocytosis (Oltrabella et al., 2015), consistent with this representing a valid therapeutic target for Lowe syndrome and Dent-2 disease. This is further supported by a study showing that chemically activating phospholipase C, which hydrolyses PI(4,5)P_2_ to IP_3_ and diacylglycerol (DAG), could rescue OCRL deficiency in both cell lines and zebrafish larvae (Mondin et al., 2019). Subsequent experiments in mice have confirmed that OCRL deficiency results in LRP2 mis-trafficking and defective endocytosis in the renal proximal tubule (Inoue et al., 2016; Festa et al., 2019), providing strong support for the original hypothesis of an LRP2 trafficking defect underlying the low molecular weight proteinuria seen in Lowe syndrome, and Dent-2 disease. Defective traffic in the endosomal pathway may also underlie other renal features of Lowe syndrome such as aminoaciduria, renal tubular acidosis, hypercalciuria and nephrocalcinosis (Bockenhauer et al., 2008). In these cases, disrupted trafficking of transporters or ion channels may occur. This would be consistent with OCRL deficiency generally impacting endosomal trafficking, as seen in *in vitro* studies (Choudhury et al., 2005; Vicinanza et al., 2011; van Rahden et al., 2012; Nandez et al., 2014), but this remains poorly explored both in zebrafish and mice.

In addition to endocytic defects in the renal proximal tubule, it was also demonstrated that OCRL mutant larvae have increased numbers and size of lysosomes (De Leo et al., 2016) (Figure 1; Table 1). Similar observations are seen in OCRL-deficient cells and renal tubules of mice (De Leo et al., 2016). Loss of OCRL causes increased lysosomal PI(4,5)P_2_ which inhibits the MCOLN1 calcium channel that is required for lysosomal fusion (De Leo et al., 2016). This reduced ability to undergo fusion results in altered lysosomal morphology and accumulation of autophagosomes in renal proximal tubule cells. Hence, defects in lysosomal homeostasis and autophagic flux may contribute to the renal tubulopathy seen in Lowe syndrome and Dent-2 disease. A similar lysosomal phenotype is seen upon IPIP27A knockout in zebrafish (Oltrabella et al., 2021), suggesting these proteins act together in maintaining lysosomal homeostasis, but the detailed mechanisms remain to be determined.

Endocytic and lysosomal defects contribute significantly to the renal pathology of Lowe syndrome and Dent-2 disease, as described above. However, other mechanisms, that may in turn impact upon the endocytic capacity of the proximal tubule, have been proposed. These relate to OCRL function in ciliogenesis and cytokinesis. Defective ciliogenesis has been reported in the proximal tubule of OCRL-deficient zebrafish larvae (Rbaibi et al., 2012) (Figure 1; Table 1), which can impact upon the functional properties of renal tubule epithelial cells. For example, ciliary signaling is important to maintain the differentiated status of these cells and their capacity to carry out endocytosis (Raghavan et al., 2014). Thus, defects in ciliary function in OCRL-deficient larvae may indirectly affect the endocytic capacity of the renal tubule. Interestingly, it was reported that the proximal tubule of OCRL mutant larvae is physically shorter compared to controls (Gliozzi et al., 2020) (Figure 1; Table 1). Because *in vitro* studies have indicated a role for OCRL in cytokinesis (Dambournet et al., 2011; Ben El Kadhi et al., 2012), it was suggested that reduced cell division could account for the reduced length of the proximal tubule in mutant zebrafish, simply due to the generation of fewer tubular epithelial cells (Gliozzi et al., 2020). Mathematical modelling suggests that such shortening of the proximal tubule could account for the reduced endocytic capacity seen in OCRL mutant zebrafish and Lowe and Dent-2 patients (Gliozzi et al., 2020).

## Use of zebrafish to identify new therapeutic strategies for Lowe syndrome and Dent-2 disease

As described above, zebrafish may be used to test potential therapeutic approaches to treat disease. It appears that approaches designed to rebalance PI(4,5)P_2_ levels in Lowe syndrome and Dent-2 disease are worth pursuing. Suppression of PIP5K activity can rescue the renal phenotype of OCRL mutant zebrafish larvae (Oltrabella et al., 2015), and a similar positive benefit is seen upon chemical activation of phospholipase C (Mondin et al., 2019). Drugs that act upon these enzymes, or that reduce PI(4,5)P_2_ abundance in other ways, would therefore seem attractive for the treatment of both conditions. Of course, altering PI(4,5)P_2_ levels could induce side effects considering the many cellular roles of this phosphoinositide species (Katan and Cockcroft, 2020), and strategies targeting these enzymes would need to be mindful of potential unwanted side effects. As well as targeted studies, zebrafish embryos and larvae are amenable to high throughout drug screening, providing a robust assay is in place that allows easy scoring of the phenotype (MacRae and Peterson, 2015; Patton et al., 2021). In this regard it is interesting to note that a novel transgenic zebrafish line has been generated that measures renal function in a quantitative and reproducible manner, which has the potential to be used in a high throughout format (Naylor et al., 2022) (Figure 2B). This reporter uses a truncated LRP2 ligand (receptor-associated protein D3 domain, RAP-D3) fused to nanoluciferase (NL), which is expressed from the liver and secreted into the bloodstream, from where it is filtered and reabsorbed into the renal tubule by endocytosis. Assessment of renal function is achieved by quantifying NL-RAP-D3 excreted into the zebrafish water using an established luminescence-based measurement protocol. The reporter can measure defects both in renal filtration, as seen upon disruption of the glomerular filtration barrier, or renal tubular absorption, as seen upon LRP2 depletion (Naylor et al., 2022). Initial experiments have shown that the reporter can reliably assess different approaches to treat Alport syndrome, a rare genetic condition that affects glomerular filtration, but so far it has not been used in a drug screen for this condition (Naylor et al., 2022). The generation of the reporter line makes phenotypic screening for Lowe syndrome and Dent-2 disease possible.

## Final considerations

The zebrafish model has proven informative for understanding the functional role of OCRL *in vivo*, as well as providing insights into the pathology of Lowe syndrome and Dent-2 disease. It can recapitulate both neurological and renal features of these conditions, which is different to the Lowe syndrome mouse model that appears to lack the neurological component of this disorder (Bothwell et al., 2011). The reason for this difference is unclear but may relate to differential splicing and expression of the functional paralogue INPP5B in mouse compared to humans and zebrafish (Bothwell et al., 2010; Bothwell et al., 2011; de Sa et al., 2025). OCRL-deficient zebrafish do not display an overt eye phenotype, although detailed characterisation has not been performed. Zebrafish have been used to model various eye pathologies including cataracts and glaucoma (Hong and Luo, 2021), and it will be interesting to perform higher resolution imaging and functional analyses of the eye in the OCRL-deficient zebrafish models. As well as providing information on disease mechanisms, the amenability of zebrafish to drug treatments and screening make it suitable for testing therapeutic strategies for Lowe syndrome and Dent-2 disease. This is particularly true with the recent development of a transgenic zebrafish reporter to quantitatively assess renal function (Naylor et al., 2022), which makes it possible to perform a phenotypic screen in OCRL-deficient zebrafish. Using a library of pre-approved drugs would enable repurposing of any ‘hit’ compounds for Lowe syndrome or Dent-2 disease, making translation to the clinic easier, faster and less expensive (Patton et al., 2021).

The genetic tractability of zebrafish is a clear benefit in using this model. However, it has been shown over recent years that chronic knockout of gene function can trigger compensatory or adaptive transcriptional responses in mutant animals, suppressing any potential phenotype (Kontarakis and Stainier, 2020; Sztal and Stainier, 2020; Cardenas-Rodriguez and Drummond, 2023). Transcriptional adaptation can occur over generations in zebrafish (Jiang et al., 2022), and epigenetic changes arising from environmental factors can also be transmitted to offspring (Cavalieri and Spinelli, 2017). Thus, one needs to be careful in the use and maintenance of chronic knockout zebrafish models. In this light, in our lab we have observed a weakening of phenotype in the OCRL-deficient genetrap line over multiple generations (unpublished). To circumvent this problem, other lines can be generated *de novo* using CRISPR-Cas9 genome editing to achieve gene knockout, or knockin of DNA into the promoter region should a hypomorphic allele akin to the genetrap be desired. Knockin of patient mutations into the OCRL gene using CRISPR-based homology-directed repair or base editing can also be performed (Liu et al., 2025). Transient approaches are also possible, and indeed, as described above, multiple studies have used morpholino knockdown to suppress OCRL expression in zebrafish, which yields a clear phenotype (Coon et al., 2012; Luo et al., 2012; Ramirez et al., 2012; Rbaibi et al., 2012; Oltrabella et al., 2015). The transient crispant approach is also now possible and can be used going forwards instead of morpholinos (Wu et al., 2018; Kroll et al., 2021), which are more prone to off-target effects (Bedell et al., 2011).

Although zebrafish has some limitations in terms of studying Lowe syndrome and Dent-2 disease, it also has many benefits, as described in this review. Used alongside other models, including the Lowe syndrome mouse (Bothwell et al., 2011; Festa et al., 2019), and patient-derived OCRL-deficient iPSC cells (Yan et al., 2021; Lo et al., 2024; Sharma et al., 2024) that can be cultured into brain or kidney organoids, it represents a valuable model going forwards. The zebrafish model complements these other models, allowing a better understanding of OCRL function *in vivo*, the mechanisms of Lowe syndrome and Dent-2 disease, and the discovery of new drug treatments for these conditions.
